# Catalytic enantioselective C(*sp*^3^)–H functionalization involving radical intermediates

**DOI:** 10.1038/s41467-020-20770-4

**Published:** 2021-01-20

**Authors:** Chi Zhang, Zhong-Liang Li, Qiang-Shuai Gu, Xin-Yuan Liu

**Affiliations:** 1 Shenzhen Grubbs Institute and Department of Chemistry, Guangdong Provincial Key Laboratory of Catalysis, Southern University of Science and Technology, 518055 Shenzhen, China; 2 Academy for Advanced Interdisciplinary Studies and Department of Chemistry, Southern University of Science and Technology, 518055 Shenzhen, China

**Keywords:** Asymmetric catalysis, Stereochemistry, Synthetic chemistry methodology

## Abstract

Recently, with the boosted development of radical chemistry, enantioselective functionalization of C(*sp*^3^)–H bonds via a radical pathway has witnessed a renaissance. In principle, two distinct catalytic modes, distinguished by the steps in which the stereochemistry is determined (the radical formation step or the radical functionalization step), can be devised. This Perspective discusses the state-of-the-art in the area of catalytic enantioselective C(*sp*^3^)–H functionalization involving radical intermediates as well as future challenges and opportunities.

## Abstract (web summary)

Enantioselective functionalization of ubiquitous C(sp^3^)–H bonds via radical species has witnessed a renaissance in the past years. Here, the authors summarize the main achievements in the field by identifying two main reaction pathways determining the stereochemistry and give an outlook on future challenges and opportunities.

## Introduction

**Fig. 1:**
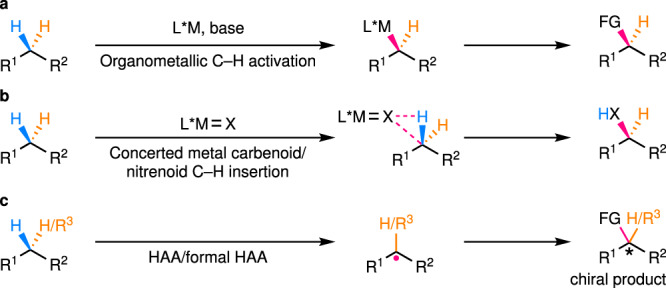
General strategies for enantioselective C(*sp*^3^)–H functionalization. **a** Transition metal-catalyzed C–H activation. **b** Concerted metal carbenoid/nitrenoid C–H insertion. **c** Sequential HAA/formal HAA and functionalization of the resulting alkyl radical. HAA hydrogen atom abstraction.

**Fig. 2:**
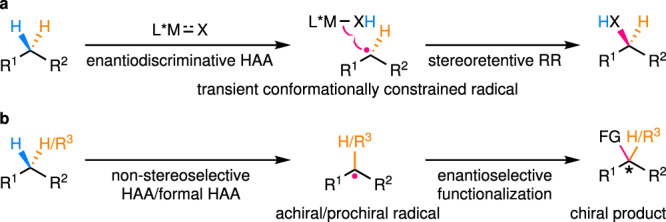
Two catalytic modes of enantioselective C(*sp*^3^)–H functionalization reactions via radical intermediates. **a** Enantiodiscriminative HAA coupled with fast stereoretentive RR. **b** Non-stereoselective HAA/formal HAA followed by enantioselective functionalization of the resulting prochiral/achiral radical. RR radical rebound.

**Fig. 3:**
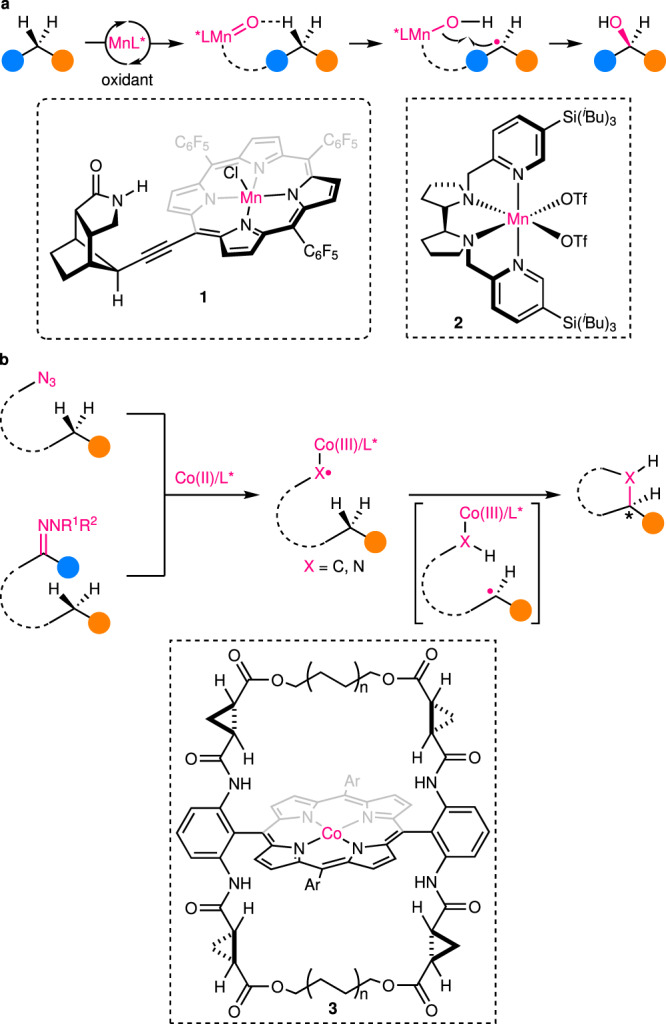
Examples of catalytic enantioselective C(*sp*^3^)–H functionalization with enantiodiscriminative HAA. **a** Mn-catalyzed enantioselective hydroxylation of secondary C(*sp*^3^)–H bonds. **b** Co-catalyzed enantioselective amination/alkylation of secondary C(*sp*^3^)–H bonds.

Direct enantioselective functionalization of C(*sp*^3^)–H bonds^1,2^, which are ubiquitous motifs in organic molecules, is undoubtedly an ideal approach to construct high value-added chiral molecules from the viewpoint of atom and step economy. In recent years, many powerful methods with different catalysts have been developed toward this goal and representative strategies include: (1) transition metal-catalyzed (typically, palladium-catalyzed) C–H activation (Fig. 1a); (2) concerted metal carbenoid/nitrenoid C–H insertion (Fig. 1b); (3) hydrogen atom abstraction (HAA) or a formal HAA process followed by functionalization of the resulting alkyl radical (Fig. 1c). With respect to the former two strategies, many excellent and comprehensive reviews have recently been published, detailing the progress, advantages, and limitations^3–5^. Reactions of the third strategy, even though they widely occur in natural biosystems, have been overlooked by chemists for a long time. This is mainly due to the highly reactive nature of radical intermediates and the lack of efficient methods to control the reactivity and enantioselectivity in radical transformations. Nonetheless, a series of elegant works on such a strategy have emerged in the past a few years, showing promising solutions to the aforementioned fundamental, yet unsolved challenge. The intention of this Perspective is to highlight some key efforts in this burgeoning research field, and thus, cannot be comprehensive. In addition, the remaining challenges and promising future directions that need to be further exploited will also be discussed. A special kind of reactions are the asymmetric cross-dehydrogenative coupling (CDC) reactions developed by Li and others^6^. Although some of them may indeed involve radical intermediates, their enantiodetermining steps usually proceed through an ionic mechanism. In addition, there have been many specialized accounts/reviews on this topic^7,8^. Thus, CDC reactions will not be covered in this Perspective. Another noteworthy kind of reactions involve the enantioselective functionalization of activated α-carbonyl C(*sp*^3^)–H bonds via radical addition to the corresponding enolate^9–12^ or enamine^13,14^ intermediates. Although significant, most of these reactions^15,16^ do not involve C–H-derived radical species and thus are out of the scope of this Perspective unless otherwise discussed below.

In essence, enantioselective C(*sp*^3^)–H functionalization reactions via a radical intermediate can be largely categorized into two catalytic modes depending on the different enantiodetermining steps. Reactions of the first catalytic mode involve initial enantiodiscriminative HAA producing a transient conformationally constrained radical, which then undergoes fast stereoretentive radical rebound (RR) before overcoming the conformational constraint imposed by its environment (solvent cage and/or catalyst cavity; Fig. 2a). In contrast, in the reactions of the second catalytic mode, a free-diffusing achiral/prochiral radical is first produced via non-stereoselective HAA/formal HAA, of which the subsequent enantioselective functionalization provides the enantioenriched product (Fig. 2b). This Perspective will be mainly organized on the basis of the classification into these two categories, with emphasis on the scope of C(*sp*^3^)–H bonds and the enantiocontrol under different catalytic modes. Definite exceptions to this classification are rare and thus, will be sporadically discussed where appropriate.

### Reactions with enantiodetermining HAA

Reactions of this first category entail both highly enantioselective HAA and sufficiently fast ensuing RR for achieving superior overall enantioselectivity. Such a mechanistic scenario is common in many enzymatic C(*sp*^3^)–H hydroxylation reactions. Specifically, substrates are believed to first undergo HAA with a high-valent metal-oxo species, and the enantioselectivity is defined by the confined cavity of enzymes, such as cytochromes P450. Next, rapid oxygen rebound between the resulting radicals and metal-hydroxy intermediates occurs with retention of the stereochemistry. Most of these enzymatic reactions have already been well summarized^17–20^, and thus will not be elaborated in this Perspective. Biomimetic catalysts of Fe and other transition metals, such as Mn and Ru with various ligands, including salen and porphyrin, have been investigated and some effective catalysts have been developed^21^. However, most of the current methods are troubled with two common problems: (1) many catalysts cannot distinguish the desired reacting C–H bond from many other ones with similar bond dissociation energies; (2) overoxidation of the resulting alcohol to ketone is often inevitable, thus diminishing the yield of the target alcohol. In regard to the first problem, Bach’s group has designed a Mn–porphyrin catalyst **1** with a structurally rigid chiral amide attachment (Fig. 3a)^22,23^. Upon exposure to substrates containing an amide moiety under oxidative conditions, the hydrogen bonds between the substrates and the catalyst direct the in situ formed high-valent Mn-oxo center to a specific (hetero)benzylic C(*sp*^3^)–H bond, thus ensuring highly regioselective and enantioselective HAA. Unfortunately, the overoxidation problem, though largely suppressed, still exists with this method. In this vein, Bietti, Costas, and coworkers have introduced a carboxylic acid into substrates to realize efficient enantioselective lactonization of unactivated C(*sp*^3^)–H bonds in adamantaneacetic acid derivatives, with chiral Mn tetradentate complex **2** as catalyst and H_2_O_2_ as oxidant (Fig. 3a)^24^. The carboxylic acid plays dual roles during the reaction: first, it coordinates with the Mn complex, providing the requisite rigidity for high regioselective and enantioselective HAA; second, it spontaneously transforms the produced alcohol to a lactone in the polarfluorinated alcohol solvent, thus efficiently avoiding the overoxidation side reaction.

Besides hydroxylation, the strategy is also applicable to enantioselective C(*sp*^3^)–H alkylation and amination reactions. In this aspect, inspired by heme enzyme-catalyzed C–H oxidation, Zhang and coworkers have designed a series of chiral Co(II)–porphyrin complexes with a well-defined open-shell doublet *d*^7^ electronic structure for metalloradical catalysis^25–30^. Upon reaction with diazo/sulfonyl hydrazone/azide precursors, these metalloradical complexes form por*–Co(III)–carbene/nitrene radicals (Fig. 3b)^31,32^, which exhibit discrete radical character for enantioselective HAA. In a latest work, a bridged chiral porphyrin ligand **3** has been utilized for the synthesis of cyclic sulfamides^33^ with excellent enantioselectivity from (hetero)benzylic, allylic, and propargylic C–H bonds, as well as other equivalents (Fig. 3b). The enantiodifferentiative HAA is supported by both kinetic isotopic effect studies and density functional theory calculations. In addition, the confined chiral cavity environment induced by the Co–porphyrin complex is shown to conformationally stabilize the facial chirality of the radical thus generated, therefore, preventing it from racemization. Interestingly, the two major HAA transition states that ultimately lead to the two enantiomeric products, respectively, are connected by the interconversion of two low-lying conformers of the reaction complex. And a short bridge length is found to be crucial for kinetically impeding this interconversion, rendering the thermodynamically favored transition state kinetically less accessible. Thus, shortening the bridge length together with installing appropriate non-chiral substituents on the arenes flanking the central porphyrin ring results in a complete reversal of the enantioselectivity, without altering the major chiral elements.

This strategy, when armed with enantioselective HAA that efficiently discriminates two enantiotopic groups, is also suitable for developing desymmetrization processes. As such, Bach’s group has disclosed highly enantiotopos-selective benzylic methylene oxidation of spirocyclic oxindole with a Ru-based catalyst structurally similar to **1** (ref. ^34^). With regard to nonactivated C(*sp*^3^)–H oxidation, Costas, Bietti, and coworkers have achieved highly enantiotopos-selective oxidation of amide-monosubstituted cyclohexane^35^. With the synergistic cooperation of a sterically demanding tetradentate Mn catalyst analogous to **2** and an oxidatively robust alkanoic acid, the reaction furnishes the corresponding chiral ketone with excellent regioselectivity and enantioselectivity. Similarly, Nam, Sun, and coworkers have employed a structurally related Mn catalyst for enantiotopos-selective benzylic C(*sp*^3^)–H oxidation of spirocyclic ketone^36^.

### Reactions with enantiodetermining radical functionalization

In reactions falling into this category, an achiral/prochiral alkyl radical is non-stereoselectively produced via direct HAA or indirect tandem electron transfer/deprotonation with or without the involvement of any chiral catalyst. In the reactions discussed above, the alkyl radicals, once generated, are immediately consumed within the solvent cage and/or ligand cavity where they are just formed. In contrast, the alkyl radicals involved in the reactions of the second category are usually free-diffusing radicals with high reactivity. Accordingly, they readily undergo racemic background reactions, evading the influence of any chiral catalyst. In addition, the usually low activation barriers for radical reactions leave any chiral catalyst with a rather limited tunable space for inducing competent energetic distribution of different stereoisomeric transition states that can ultimately lead to the favorable formation of one single enantiomer. These two facts render the enantioselective functionalization of such alkyl radicals greatly challenging. Nonetheless, a number of chiral amine, Lewis/Brønsted acid, and transition metal catalysts have recently been successfully developed, as discussed below, to accomplish a variety of enantioselective C(*sp*^3^)–H functionalization using this second catalytic mode.

### Chiral amine catalysis

**Fig. 4:**
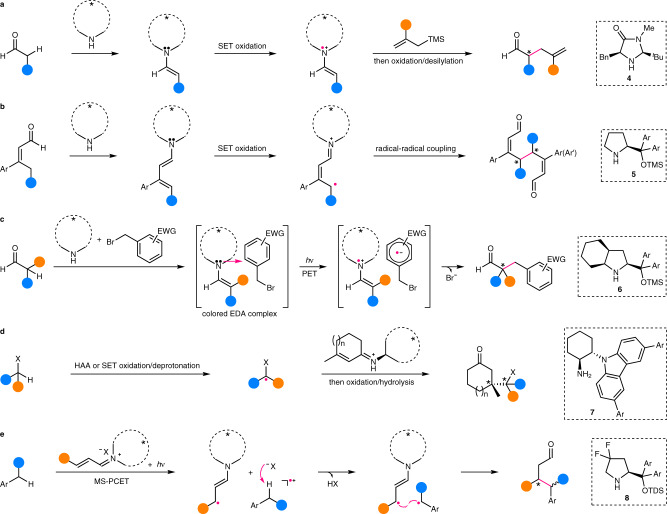
Examples of enantiocontrol by chiral amine catalysts in enantioselective C(*sp*^3^)–H functionalization. **a** Enantioselective addition of C–H-derived chiral amine-bonded prochiral radicals to free π-acceptors. **b** Enantioselective homo-/heterocoupling of C–H-derived chiral amine-bonded prochiral radicals. **c** Enantioselective coupling of C–H-derived chiral amine-bonded prochiral radicals with another type of radicals within the solvent cage of their origin. **d** Enantioselective addition of C–H-derived free achiral/prochiral radicals to chiral amine-bonded π-acceptors. **e** Enantioselective coupling of C–H-derived free achiral/prochiral radicals with another type of chiral amine-bonded radicals. TMS trimethylsilyl, EWG electron-withdrawing group, EDA electron donor–acceptor, PET photoinduced electron transfer, MS-PCET multisite proton-coupled electron transfer, TDS thexyl-dimethylsilyl.

Reactions of this type are mainly dealing with an α-C(*sp*^3^)–H bond of aldehyde via SOMO (singly occupied molecular orbital) activation, pioneered by MacMillan’s group^37^. In this regard, they have employed chiral amine **4** (Fig. 4a) to form enamine from aldehyde, of which single-electron transfer (SET) oxidation by an external oxidant leads to a 3–π-electron radical cation, i.e., a SOMO electrophile. The amine catalyst is finely tuned so that only the *si* face of this radical cation is exposed for potential reaction with a SOMO nucleophile, e.g., allylsilane^37^, thus providing a high level of enantiocontrol. In addition to enantioselective allylation, a panel of other transformations^38^ such as vinylation^39^, arylation^40^, alkynylation^41^, and alkylation^42,43^ reactions have been also established. Besides, the SOMO activation strategy has been extended to the γ-alkylation of enal via a dienamine intermediate formed by condensation with proline-derived catalyst **5**. SET oxidation of the dienamine delivers the corresponding 5–π-electron radical cation^44^, and subsequent enantioselective radical homo- or heterocoupling affords chiral bis-enal in high diastereoselectivity and enantioselectivity (Fig. 4b).

A mechanistically related α-alkylation of aldehyde has been developed by Melchiorre’s group using chiral amine **6** (Fig. 4c)^45^. In this case, a colored electron donor–acceptor (EDA) complex is preformed between the enamine and benzyl bromide, which, upon visible light irradiation, undergoes photoinduced electron transfer to provide the 3–π-electron radical cation together with a radical anion. Subsequent fast bromide extrusion and in-cage radical coupling provides the alkylation product. Noteworthy is that the configuration of the EDA complex is deemed to be largely retained during the following transformations and in this sense, the enantioselectivity is likely already determined before the radical formation. The robust reactivity of this methodology is manifested by the ready accommodation of α-disubstituted aldehyde for the challenging construction of chiral quaternary carbon stereocenters. The reaction has been extended to cyclic ketone by the same group using a sterically less demanding cinchona alkaloid-derived primary amine catalyst^46^.

In contrast to these examples using essentially the enamine catalysis, the corresponding iminium catalysis has been also maneuvered by Melchiorre’s group for enantioselective alkylation of α-heteroatom and benzylic C(*sp*^3^)–H bonds. Thus, the chiral amine **7**-derived α,β-unsaturated iminium intermediate is enantioselectively attacked by achiral or prochiral nucleophilic alkyl radicals, which are generated from corresponding acetal or amine via HAA or sequential SET oxidation and deprotonation, respectively (Fig. 4d)^47^. The advantage of radical transformations has been clearly demonstrated by the facile formation of sterically congested chiral tetrasubstituted carbon stereocenters, even two contiguous ones. As for benzylic C(*sp*^3^)–H bonds, aromatic enal, amine **8**, and an acid cocatalyst are first converted to the corresponding iminium salt, which next undergoes sequential multisite proton-coupled electron transfer with alkyl arenes upon excitation with visible light irradiation (Fig. 4e)^48^. Finally, the resulting benzylic radical is enantioselectively coupled with the accompanying chiral β-enaminyl radical.

### Chiral Lewis/Brønsted acid catalysis

**Fig. 5:**
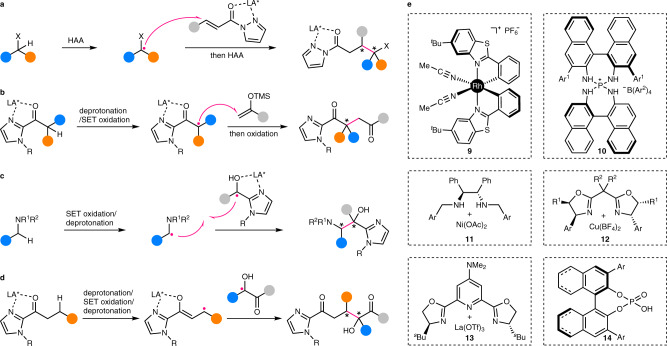
Examples of enantiocontrol by chiral Lewis/Brønsted acid catalysts in enantioselective C(*sp*^3^)–H functionalization. **a** Enantioselective addition of C–H-derived free achiral/prochiral radicals to chiral Lewis acid-coordinated π-acceptors. **b** Enantioselective addition of C–H-derived chiral Lewis acid-coordinated prochiral radicals to free π-acceptors. **c** Enantioselective coupling of C–H-derived free achiral/prochiral radicals with chiral Lewis acid-coordinated radicals. **d** Enantioselective coupling of C–H-derived chiral Lewis acid-coordinated prochiral radicals with free radicals. **e** Structures of typical Lewis and Brønsted acid catalysts employed in relevant works. LA* chiral Lewis acid.

This kind of reactions mainly covers the functionalization of activated C(*sp*^3^)–H bonds that are α to carbonyl groups or heteroatoms, such as oxygen and nitrogen. Akin to the chiral amine-catalyzed reactions discussed above, the C–H-derived alkyl radicals are enantioselectively functionalized via addition to (heteroatom-containing) double bonds or coupling with another type of in situ generated radicals, while the chiral Lewis acid catalysts are bonded to either the C–H-derived alkyl radicals or the other reactants. For example, Meggers’ group has reported enantioselective addition of nucleophilic heteroatom-stabilized (O and S) alkyl radicals generated by intramolecular HAA to α,β-unsaturated *N*-acylpyrazoles (Fig. 5a)^49^. The chiral-at-metal Rh catalyst **9** coordinates to the *N*-acylpyrazole moiety, thus rendering the conjugated alkene sufficiently more electrophilic than those in free substrates. In this way, the racemic background reaction is successfully outcompeted and high enantioselectivity is achieved. Wu’s group has later greatly extended the scope of this reaction by merging it with hydrogen atom transfer photocatalysis, thus enabling facile intermolecular HAA of aldehyde, tetrahydrofuran, amine, and 1,3-dioxolane^50^. In addition, tetrahydroisoquinoline-derived α-amino alkyl radicals has been also generated by Kang’s group via sequential SET oxidation and deprotonation for enantioselective addition to chiral Rh-coordinated 2-acyl imidazole^51^.

Similarly, the coordination of chiral Lewis acid catalysts to C–H-derived α-carbonyl alkyl radicals would also enhance their electrophilicity, thus promoting their addition to electron-rich alkenes. In this aspect, Meggers’ group has generated α-carbonyl alkyl radicals from 2-acyl imidazoles by tandem deprotonation and electrochemical SET oxidation, which subsequently add to silyl enol ethers catalyzed by a Rh catalyst analogous to **9**, affording a series of 1,4-dicarbonyl compounds with all-carbon quaternary stereocenters in high enantiopurity (Fig. 5b)^52^. As for the radical coupling scenario, the coordination of a Lewis acid catalyst to the electrophilic radicals would also boost their coupling with nucleophilic radicals. Thus, Meggers’ group has used an Ir-based analogue of complex **9** as the catalyst for achieving enantioselective coupling of nucleophilic α-amino alkyl radicals, obtained by tandem SET oxidation and deprotonation of amine, with electron-deficient ketyl radicals (Fig. 5c)^53^. Interestingly, the same group has also reported a rare direct generation of β-alkyl radicals from 2-acyl imidazole via a tandem deprotonation, SET oxidation, and deprotonation process (Fig. 5d)^54^. The subsequent enantioselective coupling with certain free α-hydroxy alkyl radicals is realized under the stereocontrol of the coordinated sterically more congested derivative of **9**. Similar enantioselective coupling of such Lewis acid-stabilized enolate radicals with simple tertiary alkyl radicals formed via intramolecular HAA of unactivated C(*sp*^3^)–H bonds has been also described by Meggers, Gong, and coworkers, and this time, a benzoxazole-based analogue of **9** is found to be a suitable catalyst^55^.

In addition to these chiral-at-metal Lewis acids, other catalysts of Ni (**11**)^56^, Cu (**12**)^57^, and La (**13**)^58^ (Fig. 5e) with various chiral ligands have recently been discovered to promote C(*sp*^3^)–H functionalization via enantioselective radical coupling. Further, chiral cationic Brønsted acid **10** (Fig. 5e) has been devised by Ooi’s group to promote the enantioselective coupling of anionic radicals with free nucleophilic α-amino alkyl radicals from SET oxidation and deprotonation of amine^59^. Both the cationic charge and the hydrogen-bond-donor ability of the catalyst are found to be essential for the reaction on the basis of a series of control experiments, supporting the indispensable association of the catalyst with the presumed anionic radical. In contrast, neutral chiral phosphoric acid (CPA) **14** (Fig. 5e) has been employed by List’s group for enantioselective coupling of α-carbonyl alkyl radicals with semiquinone, of which both are formed by PCET within the complex of hydrogen-bonded enol, CPA, and quinone^60^. Thus, α-C(*sp*^3^)–H bonds of cyclic ketones are directly oxidized with high enantioselectivity. In addition, CPA has been also briefly explored by Jiang’s group for the enantioselective coupling of C–H-derived free benzylic radicals with ketyl radicals^58^. A noteworthy feature for the reactions discussed above is the difficulty in controlling the stereochemistry on the C–H-derived alkyl radical carbons, when an amine or Lewis acid catalyst is not directly bound to these radicals. Thus, poor to moderate diastereoselectivity is usually obtained^48–51,53,55,57,58^.

### Chiral transition metal catalysis

**Fig. 6:**
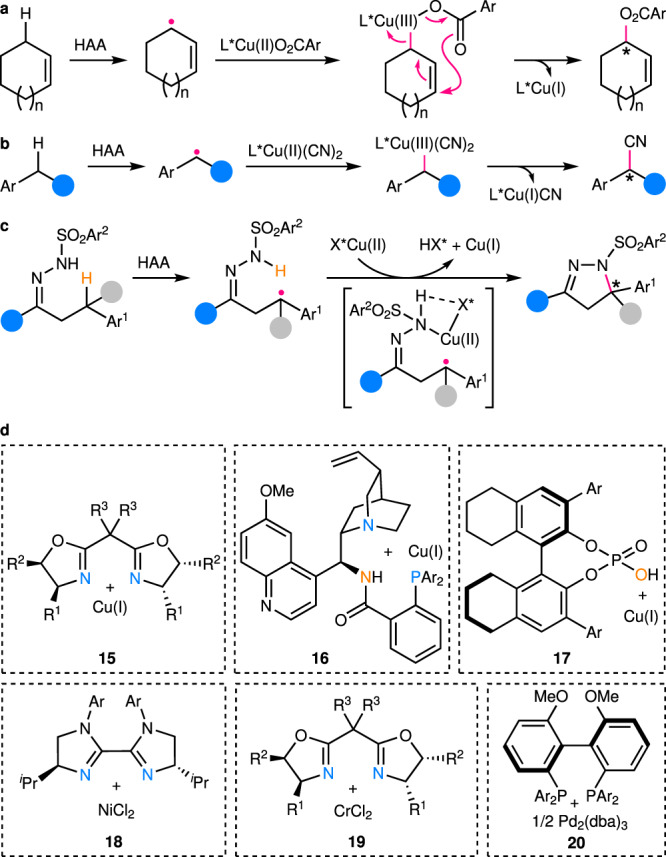
Examples of enantiocontrol by chiral transition metal catalysts in enantioselective C(*sp*^3^)–H functionalization. **a** Enantiocontrol in enantioselective Kharasch–Sosnovsky allylic C(*sp*^3^)–H acyloxylation catalyzed by chiral copper complexes. **b** Enantiocontrol in enantioselective benzylic C(*sp*^3^)–H cyanation catalyzed by copper/chiral box complexes. **c** Enantiocontrol in enantioselective tertiary benzylic C(*sp*^3^)–H amination by copper/chiral phosphoric acid dual catalysis. **d** Structures of typical chiral transition metal catalysts employed in relevant works. Box bisoxazoline.

This class of reactions mainly cover benzylic and allylic, as well as propargylic C(*sp*^3^)–H bonds, which are cleaved largely by intramolecular or intermolecular HAA. Unlike the aforementioned two types of catalysts, the redox active transition metal catalysts are able to transform reactive radical species into relatively stable polar species, thus partially alleviating the enantiocontrol challenge. For example, in the classic enantioselective Kharasch–Sosnovsky reaction (Fig. 6a)^61^, the trap of prochiral allylic radicals with chiral Cu(II) complexes leads to formation of chiral Cu(III) species, of which subsequent reductive elimination via a pericyclic rearrangement process delivers the enantioenriched allyl esters. A range of chiral ligands^61–63^, mainly oxazoline-based ones, have been developed for high enantioselectivity. Nonetheless, the substrate scope has been largely limited to simple cyclic alkenes so far.

A breakthrough in this area has recently been made by Liu, Stahl, and their coworkers with the discovery of a highly efficient and enantioselective intermolecular cyanation of benzylic C(*sp*^3^)–H bonds (Fig. 6b), using the Cu(I)/chiral bisoxazoline (box) catalyst **15** (Fig. 6d)^64^. In this case, prochiral benzylic radicals are generated via HAA by arylsulfonimidyl radicals and next are trapped by chiral Cu(II) complexes to afford Cu(III) intermediates. Direct reductive elimination from these intermediates leads to enantioenriched α-chiral nitriles with a broad substrate scope. DFT calculations support reversible radical trap and subsequent enantiodetermining reductive elimination. In addition, the reaction has been extended to intermolecular benzylic C(*sp*^3^)–H arylation^65^ and alkynylation^66^ by replacing the cyanation reagent TMSCN (trimethylsilyl cyanide) with aryl boronic acid and alkynyl silane, respectively. Furthermore, the groups led by Liu, Nagib, Wang, and Yu have independently reported enantioselective benzylic C(*sp*^3^)–H cyanation^67–70^ and alkynylation^71^ by merging oxidative radical precursors into substrates, which upon SET reduction provides alkoxy, sulfonamidyl, or amidyl radicals for intramolecular 1,5-HAA with high regioselectivity. At the same time, Liu’s group has also found mildly oxidative *N*-fluoroamide is well compatible with their Cu(I)/cinchona alkaloid-derived N,N,P-ligand catalyst **16** (Fig. 6d)^72–77^, which readily reduces *N*-fluoroamide to amidyl radicals for highly regioselective intramolecular 1,5-HAA under ambient conditions^78^. Accordingly, highly regioselective and enantioselective benzylic C(*sp*^3^)–H alkynylation is achieved with terminal alkynes, of which the undesired Glaser homocoupling is efficiently suppressed. The Cu(I)/chiral box catalyst system has been also utilized by Liu’s and Lin’s groups for highly site- and enantioselective intermolecular allylic C(*sp*^3^)–H cyanation^79^. The site-selectivity is postulated to be due to HAA by Cu-bound sterically demanding sulfonamidyl radicals, which is supported by both mechanistic experiments and theoretical calculations. Notably, this catalytic system has just been reported by Nagib’s group for enantioselective C(*sp*^3^)–H amination with the enantiocontrol over both the intramolecular HAA by a chiral Cu(II)-bound imidate radical and the subsequent C–N formation step^80^. The robustness of such a mechanistic scenario has been shown by the accommodation of even unactivated C(*sp*^3^)–H bonds. Interestingly, synergistic enantiocontrol over two tandem SET oxidation/deprotonation and HAA processes by chiral phosphate and chiral thiol catalysts, respectively, has also recently been disclosed by Knowles, Miller, and coworkers for deracemization of urea^81^.

Another breakthrough has been the achievement of enantioconvergent functionalization of racemic tertiary C(*sp*^3^)–H bonds (p*K*_a_ > 25) by Arnold’s and Liu’s groups using mutated enzymes^82^, and Liu’s group using Cu(I)/CPA catalysts^83–85^, respectively. The challenge for developing such a transformation mainly rests on the issue of chirality retention, which is common to most of the C(*sp*^3^)–H functionalization methods based on the transition metal-catalyzed C–H activation, the concerted metal carbenoid/nitrenoid C–H insertion, or the coupled enantiodiscriminative HAA/stereoretentive RR mechanistic manifolds. Thus, only kinetic resolution of racemic tertiary C(*sp*^3^)–H bonds is, in theory, likely achievable using these methods^86,87^. To this end, Arnold, Liu, and coworkers have managed to identify a mutated variant of cytochromes P441 (ref. ^82^), designated as P441_Dianel3_, catalyzing the enantioconvergent intramolecular amination of racemic tertiary benzylic C(*sp*^3^)–H bonds with sulfamoyl azide. Of particular note is that another variant P441_Dianel4_ even fulfills the rare enantioconvergent amination of an unactivated tertiary C(*sp*^3^)–H bond bearing a methyl and an ethyl substituent. The DFT calculations on a model system suggest a relatively slow RR process that allows for facile conformational rotations of the radical intermediates, leading to complete loss of the original stereochemical information in advance. To address the same challenge with transition metal catalysts, Liu’s group has deliberately combined their chiral Cu(I)/CPA catalysis (**17** in Fig. 6d) with a decoupled intramolecular 1,5-HAA by in situ generated sulfonyl hydrazonyl radicals (Fig. 6c)^83^. The HAA process does not involve any copper species and thus, the loss of stereochemical information on the generated alkyl radicals before their subsequent functionalization is expected. Subsequent association of the thus-obtained prochiral tertiary benzylic radicals with Cu(II)/chiral phosphate leads to the enantioselective C–N bond formation. In addition, Liu’s group has employed the same catalyst system for realizing enantioselective intramolecular prochiral benzylic and allylic C(*sp*^3^)–H amination^88^. In this case, an *N*-hydroxyphthalimide derivative is found to be indispensable as a mediator for achieving highly efficient intermolecular HAA.

In addition to copper catalysts, a Ni/chiral biimidazoline catalyst **18** has been reported by Lu’s group for enantioselective benzylic C(*sp*^3^)–H arylation reaction with aryl bromides (Fig. 6d)^89^. According to the mechanistic experiments, the benzylic C(*sp*^3^)–H is likely abstracted by a photochemically generated bromine radical, and the resulting prochiral benzylic radical is then captured to form a chiral Ni(III) complex. Subsequent reductive elimination from this complex affords the biologically important chiral 1,1-diaryl alkane products. Unfortunately, excess C–H substrates (4 equivalents or more) are necessary for obtaining satisfactory yields.

A Cr/chiral box catalyst **19** has been also disclosed by Mitsunuma, Kanai, and coworkers for enantioselective allylation of aldehyde via allylic C(*sp*^3^)–H functionalization (Fig. 6d)^90^. An achiral/prochiral allylic radical is first produced via a tandem SET oxidation/deprotonation process in the presence of a strong oxidative acridinium photocatalyst under visible light irradiation. Interception of the allylic radical by the Cr(II)/chiral box catalyst **19** gives rise to the key chiral allylic Cr(III) species, which reacts with the aldehyde likely via a six-membered cyclic transition state to provide the enantioenriched allylation product. Currently, only cyclic disubstituted and acyclic multi-substituted alkenes with relatively low oxidation potentials are applicable to this reaction.

In addition to the functionalization of these π-activated C(*sp*^3^)–H bonds, enantioselective alkylation of α-amino C(*sp*^3^)–H bonds has just been demonstrated by Yu’s group using a Pd/chiral bisphosphine catalyst **20** (ref. ^91^). The α-amino alkyl radicals are first formed by sequential SET oxidation and deprotonation, which next coordinate to in situ generated chiral allyl Pd(II) complexes to forge Pd(III) intermediates. Lastly, reductive elimination of these high-valent intermediates leads to enantioenriched α-allylated aniline products.

### Conclusion and future perspectives

Enormous strides in the area of enantioselective C(*sp*^3^)–H functionalization involving radical intermediates have been made during the past years, providing fresh opportunities in synthetic radical chemistry, and therefore stimulating more endeavors in this burgeoning field. As briefly summarized in this Perspective, these nascent yet powerful methods, which generally fall into two categories distinguished by their different stereo-determining steps, have been exploited to give enantioenriched products from relatively simple C(*sp*^3^)–H substrates without pre-functionalization. Some of them have been also shown to be applicable to complex natural products and drug molecules, showcasing their potentials in late-stage functionalization. However, major developments discussed as follows are still necessary for the broad application of these methods.

**Fig. 7:**
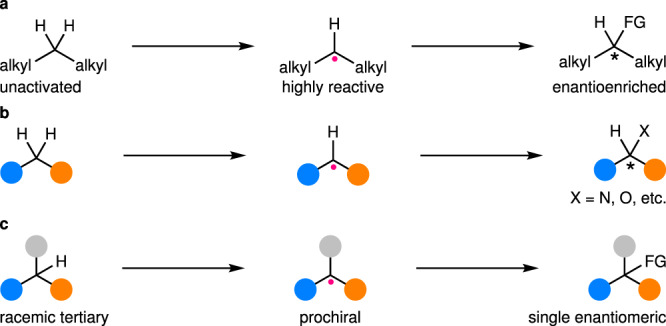
Ongoing major challenges for enantioselective C(*sp*^3^)–H functionalization via radical intermediates. **a** Enantioselective functionalization of unactivated C(*sp*^3^)–H bonds without any directing/stabilizing remote substituent. **b** Enantioselective functionalization of C(*sp*^3^)–H bonds with various heteroatom-based functionalities. **c** Enantioconvergent functionalization of racemic tertiary C(*sp*^3^)–H bonds, ideally in an intermolecular manner.

First, enantioselective functionalization of unactivated C(*sp*^3^)–H bonds for the construction of stereocenters on the original carbons has been a daunting task (Fig. 7a). In fact, they are generally more abundant than activated ones in common commercially available starting materials, natural products, drugs, and bioactive small molecules. Thus, realizing such a transformation would be highly desirable. Nonetheless, the corresponding alkyl radicals are much more reactive than those derived from activated C(*sp*^3^)–H bonds due to the lack of stabilizing interactions. This has rendered the catalysts in the second catalytic mode category inapplicable so far, with the sole exception of enzymes^82^. In this aspect, intramolecular directing groups have been introduced to facilitate the trap of such radicals by chiral transition metals^92–97^, realizing their enantioselective functionalization. However, the corresponding enantioselective C(*sp*^3^)–H functionalization using such a strategy remains rare^80^. In contrast, for the catalysts in the first catalytic mode category, the enantioselectivity has been determined during the radical generation, and thus, the problem seems not be exceptionally severe as long as the stereoretentive RR is maintained. Nonetheless, the corresponding successful examples are still rare^24,80^.

Secondly, enantioselective functionalization of C(*sp*^3^)–H bonds with heteroatoms remains much less established compared to that involving C–C bond formation (Fig. 7b). Heteroatom-based functionalities, particularly amines and alcohols, are important elements that decorate the carbon skeletons in a number of chiral bioactive or functional molecules. In addition, they also constitute excellent starting functionalities for further derivatization to increase molecular complexity. However, most of the known methods for their installation into C(*sp*^3^)–H bonds via alkyl radical intermediates are either of limited efficiency and/or substrate scopes^21^ or confined to intramolecular reactions^29,30,33,80,82,83,88^. Accordingly, continued endeavors toward highly efficient intermolecular hetero-functionalization of C(*sp*^3^)–H bonds are still in high demand.

Thirdly, catalytic enantioconvergent functionalization of racemic tertiary C(*sp*^3^)–H bonds (p*K*_a_ > 25) remains largely underdeveloped (Fig. 7c). The resulting chiral tetrasubstituted carbon stereocenters are important motifs in many natural products and pharmaceuticals. Currently, the only workable strategy is using chiral transition metal catalysis for enantioselective functionalization of prochiral tertiary alkyl radicals. Nonetheless, successful examples are limited to intramolecular amination^82,83^. In fact, transition metal-catalyzed enantioselective intermolecular functionalization of tertiary α-carbonyl alkyl radicals has started to emerge^98–100^, which should encourage more efforts toward implementing enantioselective intermolecular functionalization of racemic tertiary C(*sp*^3^)–H bonds (p*K*_a_ > 25).
